# A lightweight hybrid vision transformer network for radar-based human activity recognition

**DOI:** 10.1038/s41598-023-45149-5

**Published:** 2023-10-21

**Authors:** Sha Huan, Zhaoyue Wang, Xiaoqiang Wang, Limei Wu, Xiaoxuan Yang, Hongming Huang, Gan E. Dai

**Affiliations:** 1 School of Electronics and Communication Engineering, Guangzhou University, Guangzhou, 510006 China; 2 Key Laboratory of On-Chip Communication and Sensor Chip of Guangdong Higher Education Institutes, Guangzhou, 510006 China; 3 College of Naval Architecture and Ocean Engineering, Naval University of Engineering, Wuhan, 430033 China; 4 School of Electronic Information Engineering, Foshan University, Foshan, 528225 China

**Keywords:** Engineering, Electrical and electronic engineering

## Abstract

Radar-based human activity recognition (HAR) offers a non-contact technique with privacy protection and lighting robustness for many advanced applications. Complex deep neural networks demonstrate significant performance advantages when classifying the radar micro-Doppler signals that have unique correspondences with human behavior. However, in embedded applications, the demand for lightweight and low latency poses challenges to the radar-based HAR network construction. In this paper, an efficient network based on a lightweight hybrid Vision Transformer (LH-ViT) is proposed to address the HAR accuracy and network lightweight simultaneously. This network combines the efficient convolution operations with the strength of the self-attention mechanism in ViT. Feature Pyramid architecture is applied for the multi-scale feature extraction for the micro-Doppler map. Feature enhancement is executed by the stacked Radar-ViT subsequently, in which the fold and unfold operations are added to lower the computational load of the attention mechanism. The convolution operator in the LH-ViT is replaced by the RES-SE block, an efficient structure that combines the residual learning framework with the Squeeze-and-Excitation network. Experiments based on two human activity datasets indicate our method’s advantages in terms of expressiveness and computing efficiency over traditional methods.

## Introduction

Human activity recognition (HAR) has huge potential for numerous applications, such as intelligent healthcare, smart homes, intelligent security, and autonomous driving. In recent years, HAR data sources have been categorized into two groups: visual-based HAR and non-visual sensor-based HAR^1^.Visual-based HAR^2^ analyzes human motion using video or photos acquired by optical cameras, whereas non-visual sensor-based HAR collects data using smart sensors^3^ such as gyroscopes, accelerometers, and radars. Millimeter-wave radar can adapt to different weather and lighting conditions with low power consumption and privacy protection. Considerable attention has been paid to HAR technology based on millimeter-wave radar^4,5^.

Time-varying kinematic information integrating human motion^6^ can be investigated by analyzing and processing millimeter-wave radar echo signals, and activity recognition may be carried out utilizing the resulting kinematic information. Radar-based HAR is usually based on the micro-Doppler feature of target echoes. Micro-Doppler features from the time-Doppler graph can highlight the self-vibration and rotation of the human’s torso and limbs. Based on the clear and unique correspondence between the micro-Doppler features and human behaviour, supervised learning methods are usually used for radar-based HAR. However, HAR methods with high accuracy and embeddable potential are facing challenges and it is worthwhile devoting much effort to this.

Traditional classification techniques such as multi-layer perceptron, principal component analysis (PCA), support vector machines (SVM)^7^ and linear discriminant analysis are used in some research. Manually extracted micro-Doppler characteristics are typically employed as classification inputs^8–10^. Prior knowledge and the intricacy of the categorization task restrict the use of these characteristics. Deep learning has been steadily advancing in recent years, its excellent categorization performance has also garnered considerable attention. Radar-based HAR research has gotten more intelligent due to the in-corporation of deep learning (DL) techniques. Convolutional neural networks (CNN)^11^, re-current neural networks (RNN)^12^, transformers^13^, and hybrid networks^14^ are the four broad classifications of DL techniques. These methods use supervised learning to automatically extract sample features, hence overcoming the limitations of conventional models for feature extraction. Using recursive neural networks, time-series models can extract temporal correlation characteristics between data sequences. Numerous studies have demonstrated that adding long short-term memory (LSTM)^15^ and Bi-directional long short-term memory (BiLSTM)^16^ architectures to a network can effectively enhance HAR’s recognition performance. Furthermore, LSTM and BiLSTM was combined to achieve HAR^17^. Multi-layer BiLSTM^18^ was used to classify human activities with an average accuracy of around 90%. However, the large number of parameters of the networks above will be a computational burden in embedded applications. Lightweight CNN^19,20^ was utilized to reduce the number of parameters and improve running performance substantially, but at the cost of missing some details, resulting in a decline in recognition accuracy.

By combining the strengths of the constituent networks, hybrid networks such as CNN-LSTM^21^ can outperform single networks. The spatiotemporal features of the input data can be completely exploited by this hybrid structure and improve recognition precision. Inspired by the attention process, researchers have combined attention modules with neural networks for various purposes^22,23^. Typically, attention modules are not utilized alone, they are incorporated into various neural networks to increase network performance. Attention methods were added into residual networks, convolutional auto-encoders and LSTM. Networks with attention mechanisms achieve faster convergence and greater recognition accuracy. Attention typically avoids the problem of disappearing gradients because it provides direct links between all data time steps. In contrast to convolutional neural networks, which must preserve spatial locality in the input data, self-attention mechanisms can process data at any place in the input sequence. This increases the generalizability of the network while processing radar images of various sizes, shapes, and orientations.

The Transformer network drops the usual neural network calculation method in favour of self-attention methods for network calculation. The vision transformer (ViT)^24^ has performed extraordinarily well in the field of vision because of its usage of attention mechanisms. However, the majority of ViT networks include a large number of parameters and are challenging to implement in embedded applications. In recent years, some lightweight ViT^25–27^ structures were proposed to reduce the number of parameters while maintaining precision. However, more in-depth work on the lightweight of ViT is worth looking forward to.

Considering the embedded application background of radar-based HAR, some work has attempted to solve the efficiency and performance issues^28,29^, but new networks need to be developed to improve the recognition performance on the lightweight structures more effectively. To achieve high-accuracy HAR, this paper developed a lightweight hybrid Vision Transformer (LH-ViT) network. The network uses the residual structure joint Squeeze-and-Excitation (SE) module (RES-SE) block to form a feature pyramid for HAR feature extraction at different scales. The following stacked RadarViT networks are designed to enhance useful features through self-attention. The radar data in different bands verify that LH-ViT can achieve efficient HAR at different Doppler scales. Moreover, the LH-ViT employs depthwise separable convolution and lightweight attention models, which greatly reduce the parameter count compared to conventional ViT while maintaining the same level of accuracy.

The contributions of our research are summarized as follows:

1. We developed a novel lightweight hybrid Vision Transformer (LH-ViT) in this paper. LH-ViT combines a feature extraction network with a pyramid structure and a feature enhancement network consisting of stacked Radar-ViT components. The primary innovation of LH-ViT lies in its ability to enhance the representational power of radar-based HAR effectively by incorporating spatial attention into the micro-Doppler feature hierarchy. We conducted an in-depth investigation to optimize the structure of this proposed network. Furthermore, we conducted a comprehensive comparison of LH-ViT with several state-of-the-art HAR networks, using both our self-established dataset and a publicly available dataset”.
2. An efficient RES-SE block is designed to replace the traditional convolution operator. Operating within a residual learning framework, the RES-SE module employs depthwise separable convolutions to extract micro-Doppler features with reduced computational overhead. The lightweight SE module is inserted in the RES-SE block, which adaptively adjusts feature channel weights for enhanced representation accuracy.
3. Radar-ViT is developed as a lightweight design of ViT, which enables embedded applications of transformer-based models. Radar-ViT simplifies the traditional class token module to a point-wise convolution. Additionally, we introduce fold and unfold operations to reduce the computational demands of the multi-head attention block, prioritizing essential micro-Doppler features. Stacked Radar-ViTs excel at capturing global features on the micro-Doppler map, resulting in superior HAR performance.

The remainder of the paper is organized as follows. Section “Radar-based HAR with LH-ViT” introduces the structure and key modules of the proposed LH-ViT network. Section “Experimental results” provides the experimental findings of two datasets to validate the proposed algorithm’s superiority. Finally, Section “Conclusion” presents the conclusions.

## Radar-based HAR with LH-ViT

**Figure 1:**
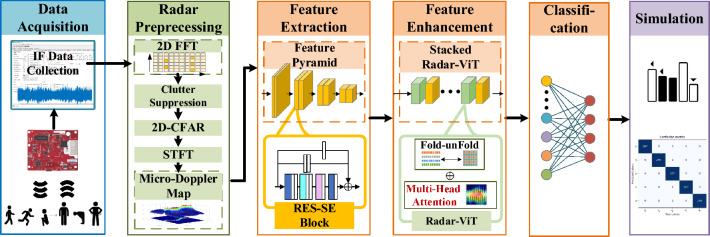
The framework of radar-based HAR with LH-ViT.

Figure 1 shows the framework of radar-based HAR with LH-ViT in this section. The millimeter wave radar collects the echo from the moving human body and outputs multi-channel intermediate frequency signals after dechirp processing. The multi-channel intermediate frequency signals are first preprocessed with 2D FFT. 2D FFT processing compresses the signal energy at the corresponding position on the range-angle plane. A phase average cancellation method^29^ is then utilized for the static clutter suppression, which will preserve the micro-Doppler signal components. Two-dimension constant false alarm rate (2D-CFAR) is applied to detect the target against the noise background. After the target bin is locked, the target bins on the range-angle plane from each frame are combined into a slow-time vector. This vector is transformed by the short-time Fourier transform (STFT) to generate MDM that reflects the target’s motion in the time-frequency domain. The normalized MDM is fed into the subsequent LH-ViT network for high-efficiency HAR. The LH-ViT is composed of a feature extraction network, a feature enhancement network, and a classification module. Maximum pooling and linear layers are used in the classification module to output the prediction results. The specific implementation of the first two networks will be introduced in the following subsections.

### Feature extraction network

**Figure 2:**
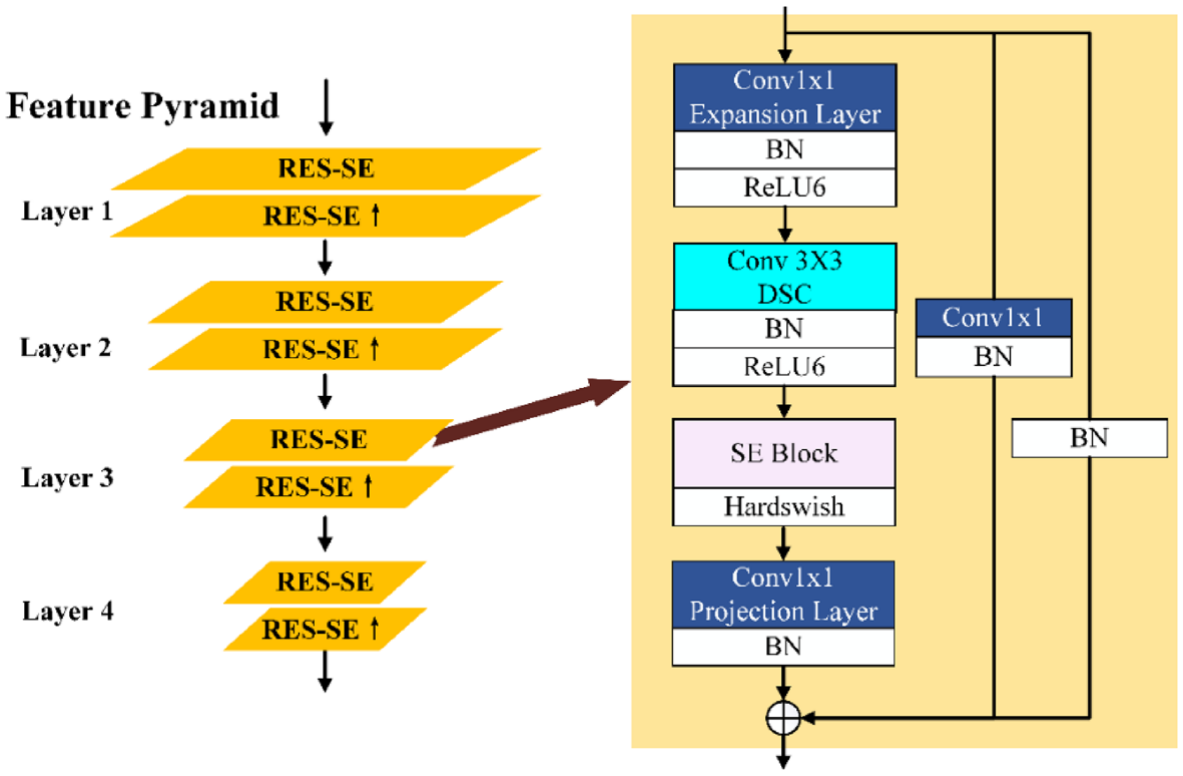
Feature extraction network structure diagram.

A pyramid structure is adopted in the feature extraction network, as shown in Fig. 2. The feature pyramid can capture the multi-scale micro-Doppler feature on the MDM. Especially when the Doppler range is relatively large and the micro-Doppler expressions are compressed, the network can still learn the activity features from the MDM accurately and effectively. In terms of a specific implementation, each layer of the pyramid uses a pair of RES-SE modules to achieve efficient feature extraction. In each layer, the first RES-SE module is used for the micro-Doppler feature extraction at the current scale, and the second RES-SE module realizes upsampling by adjusting the stride value.

**Figure 3:**
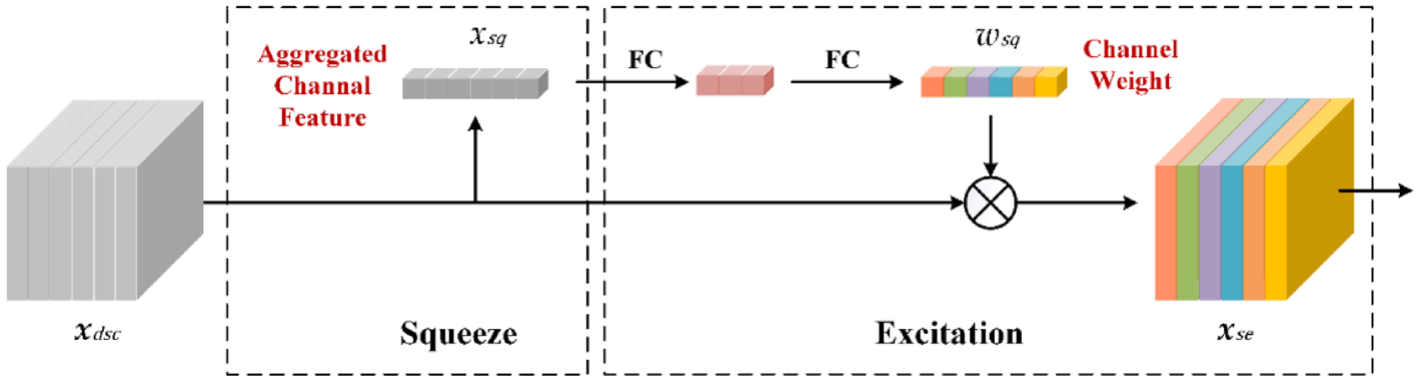
Schematic of the Squeeze-Excitation (SE) block.

The RES-SE module applies a residual network structure to achieve feature fusion at different levels. Two branches are added, performing 1 × 1 convolution joint BN operation, and only BN operation respectively. The backbone of RES-SE uses 1 × 1 convolution for dimension expansion and then uses 3 × 3 Depthwise separable convolution (DSC)^30^ for first-level feature extraction. DSC is an effective approach for the lightweight design of standard convolution operations. DSC improves on the standard convolution by decomposing it into depthwise convolution and point convolution. As a representative of a lightweight network, DSC can achieve feature extraction with lower parameter amounts and computational costs. Subsequently, an SE Block^31^ based on a light-weight channel attention mechanism is used to process the output of DSC, as shown in Fig. 3.

The output of DSC is the local spatial correlation obtained by the 2D spatial kernel. The channel dependencies are implicitly embedded in each channel of the DSC output, entangled with the spatial features. The SE block achieves inter-channel attention in a lightweight structure by explicitly modeling the channel dependencies, thereby enhancing the feature sensitivity in the channel dimension.

First, the squeeze module uses global average pooling to arrogate each 2D channel into a channel descriptor.

1 $${x}_{sq}=\frac{1}{H\times W}\sum_{i=1}^{H}\sum_{j=1}^{W}{{\varvec{x}}}_{dsc}(i,j).$$

${{\varvec{x}}}_{dsc}$ is denoted as the DSC output with $H\times W$ spatial dimension. As the channel descriptor, ${X}_{sq}$ is a statistical parameter, which represents the aggregated feature of the current channel. All the ${X}_{sq}$ are processed through a bottleneck structure consisting of two fully connected layers and a sigmoid activation in the excitation module. This bottleneck structure can capture the inter-channel dependencies flexibly. A channel dimensional adjusting rate of 4 is adopted in this work. After the excitation module obtains the weights of the different channels ${w}_{sq}$ according to their importance, a weighting process is performed on the corresponding channels. The SE Block achieves channel adjustment with fewer parameters through refined model design, emphasizing the channels with more separable information, and suppressing channels less useful.

2 $${{\varvec{x}}}_{se}={w}_{sq}{{\varvec{x}}}_{dsc}$$

After the channel attention processing in the SE block, the backbone features are projected through a 1 × 1 convolution and combined with the two branch results to obtain a more effective high-dimensional expression of micro-Doppler behaviour features.

Each 1 × 1 convolution and DSC operation are followed by a Batch normalization (BN) layer and a non-linear activation function ReLU. The BN layer implements normalization by calculating the mean and variance of the input. A Hardswish activation function is used to process the output of the SE block. The nonlinearity of the Hardswish is defined as

3 $$\mathrm{hardswith}\left[x\right]=\frac{x\mathrm{ReLU}6(x+3)}{6}.$$

It has been verified that it performs better in the deeper network. The Hardswish can reduce the filter number under the same precision.

### Feature enhancement network

The feature extraction network focuses on the local micro-Doppler feature extraction at different scales. The feature enhancement network can eliminate background noise interference effectively^32^ and highlight the micro-Doppler features related to human behavior based on multi-scale feature extraction. In this paper, cross-stacked Radar-ViT and RES-SE modules are applied to achieve global feature enhancement. In the combination structure, the RES-SE module learns the local representation of the micro-Doppler features with spatial inductive bias. The Radar-ViT processes the global information encoding of the HAR. This hybrid structure enables us to design a shallow and narrow lightweight network.

**Figure 4:**
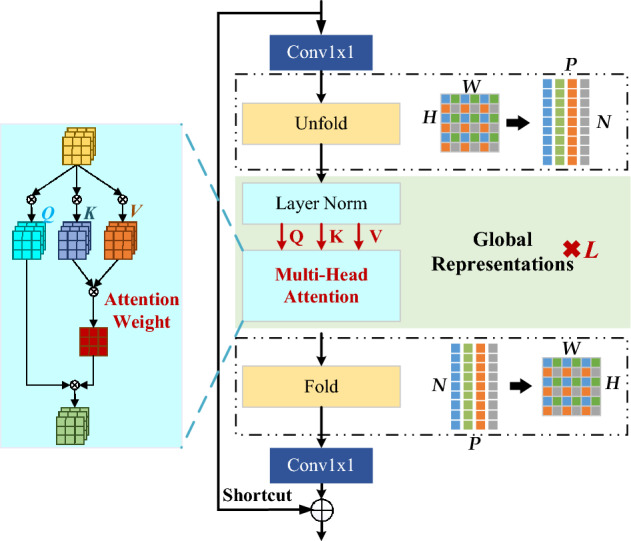
Radar-ViT diagram based on multi-head attention mechanism.

Considering the RES-SE modules at both ends, Radar-ViT further simplifies the local representation and fusion modules of MobileVit, as shown in Fig. 4. Two 1 × 1 convolutions are designed around the stacked global representation modules for the channel adjustment, to keep the consistent scales of the input and output.

Assuming that the size of the feature map is $H\times W$, the feature map of each channel is divided into non-overlapping cells of size $P$, with the total number of $HW/P$. The unfold operation after the point-wise convolution downsamples each feature map to form $P$ non-overlapping flatten patches. The position information within each cell is retained between the $P$ flatten patches, and the spatial relationship between the cells, that is, the global micro-Doppler features is preserved in each flatten patch. Therefore, although the subsequent multi-head attention modules act on the downsampled flat patch, the overall effective receptive field is $H\times W$. Multi-head attention is the key module in ViT, which is a combination of multiple self-attention blocks. The input is linearly mapped through learnable matrixes into three variables with the same dimension, namely query ${\varvec{Q}}$, key ${\varvec{K}}$, and value ${\varvec{V}}$. The normalized similarity between ${\varvec{Q}}$ and ${\varvec{K}}$ is used as the weight of ${\varvec{V}}$. The self-attention model adopts the short-cut structure from the residual network, which can effectively prevent the degradation problem. The feature outputs from different attention heads are combined by a Concat. Multiple heads enable the network to capture abundant feature information from different representation subspaces.

4 $$\mathrm{Attention}\left({\varvec{Q}},{\varvec{K}},{\varvec{V}}\right)=\mathrm{softmax}\left(\frac{{\varvec{Q}}{{\varvec{K}}}^{\mathrm{T}}}{\sqrt{{d}_{k}}}\right){\varvec{V}}.$$

Radar-ViT obtains a global representation of the micro-Doppler feature within each flatten patch separately by $L$ stacked normalization modules and multi-head attention modules. The global micro-Doppler feature can restore its scale through the fold operation. After a point-wise convolution, the fold output is combined with the Radar-ViT’s input via concatenation operation. The shortcut branch provides another direct path, allowing faster information propagation. It can accelerate the training process, speed up model convergence, and enhance recognition accuracy. These concatenated features are fused in the subsequent RES-SE modules.

## Experimental results

### Experiment dataset

Two datasets were used to validate the superiority of the LH-ViT. The public dataset is collected by a C-band radar^33^. The radar’s working bandwidth is 400 MHz. The chirp period is 1 ms. This dataset contains radar echoes of 6 human activities. Among them, 5 human activities were collected with a duration of 5 s, namely sitting in a chair, standing up, bending to pick up an object, drinking from a cup or glass, and falling. The collection time of the walking activity is 10 s. Due to the lack of data corresponding to the falling activity, the experiment only uses the other five human activity data in this paper. The sketch images, MDMs, and quantities of different human activities in the public datasets are listed in Table 1. The experiments on the public dataset are measured at 656 × 656.

**Table 1:** The public dataset collected by a C-band radar.

Label	(0)	(1)	(2)	(3)	(4)	(5)
Sketch map						
Activity	Walking back and forth	Sitting in a chair	Standing up	Bending to pick up an object	Drinking from a cup of glass	Falling
MDM						
Quantity	312	312	311	311	310	198

The self-established dataset developed by Guangzhou University is collected by a millimeter wave (mmWave) radar working at 79 GHz. The mmWave Radar’s working bandwidth is 3.68 GHz. The chirp period is 392 μ $\mathrm{s}$. The experiment was carried out in a laboratory. The radar platform was 1.5 m in height. The self-established dataset contains the radar echo data of 5 human activities. They are walking, running, standing up after squatting down, bending, and turning respectively. The self-established dataset collects the human activities of 10 participants, including 7 males and 3 females. To increase the within-class diversity of this dataset, the participants varied in age, height, and weight. Radar data were recorded according to their respective behavior habits, with no special behavior constraints attached. To expand the data amount, data augmentation was applied additionally only to the self-established dataset. The sketch images, MDMs, and quantities of different human activities in the public datasets are listed in Table 2. The experiments on the self-established dataset are measured at 224 × 224.

**Table 2:** The self-established dataset collected by a mmWave radar.

Label	(0)	(1)	(2)	(3)	(4)
Sketch map					
Activity	Walking	Running	Standing up after squatting down	Bending	Turning
MDM					
Quantity	990	990	990	990	990

Both datasets were divided into 80% for training and 20% for testing at random. MATLAB is applied for the radar signal processing of MDM. PyTorch 11.3 is used to build a DL model. The adaptive moment estimation (Adam) optimizer is utilized for network training. The learning rate is set to 0.0001. A dropout with a probability of 0.5 is applied after each pyramid layer. All the experiments in this paper are based on a hardware platform with an Intel i9 16-core CPU and one NVIDIA 3090 24G GPU.

### Network structure discussion

The LH-ViT network proposed in this work consists of a multi-layer pyramid and alternate stacked Radar-ViT and RES-SE models. The recognition performance and efficiency of the LH-ViT are closely related to the number of the pyramid layer, the alternate stacked Radar-ViT and RES-SE models. A trade is essential between the feature representation and the computational efficiency of the LH-ViT. The feature representative capability can be enhanced along with the deepening of the network for the raised nonlinear expression ability. Deep networks are capable of fitting more complex features. However, performance saturation, optimization difficulties, and shallow learning decline also occur as the network deepens. The test results on the network structure in this section are all based on the self-built dataset.

First, the optimal massive structure is determined by different combinations of the pyramid layers, the Radar-ViT, and the RES-SE stacking number. $L$ in each Radar-ViT is fixed as 2. The HAR average accuracy, the parameter quantity, the floating point operations (FLOPs), and the inference time are used as the indicators of the network performance.

Based on the self-established dataset, Table 3 discusses the optimal structure of the proposed LH-ViT network. This table also includes the ablation experiment. For concise structure representation, $i-j-k$ is used to indicate that the feature extraction part of the network structure contains $i$ level pyramids, and the feature enhancement part contains $j$ Radar-ViT and $k$ auxiliary RES-SE modules. In general, the accuracy of the LH-ViT increased along with the deepening of the network structure. But when the number of pyramid layers rises to more than 4 layers, the deeper structure contributes little to the network performance. Taking the LH-ViT(4-2-1) as an example, it is the smallest structure with an accuracy greater than 99%. This structure achieves 99.7% HAR accuracy with a parameter amount of 769.32 K. When the pyramid layers number rises up to 5 with the rest of the structure unchanged, the parameter amount increases by 176.576 K, but the recognition accuracy rate decreases by 0.2%.

**Table 3:** Discussion of the LH-ViT network structure based on the self-established dataset.

Pyramid layer	Radar-ViT number	RE-SES number	Parameters	FLOPs	Inference time	Accuracy
0	2	1	124.464 K	1.56 G	24.29 ms	91.7%
1	1	1	274.888 K	0.87 G	6.86 ms	93.9%
2	1	1	288.092 K	0.92 G	4.07 ms	95.7%
3	0	0	101.200 K	0.34 G	3.82 ms	93.2%
3	1	1	344.948 K	1.09 G	3.75 ms	97.9%
3	2	1	299.224 K	0.96 G	5.06 ms	97.5%
3	2	2	361.464 K	1.15 G	6.05 ms	98.2%
4	0	0	146.488 K	0.48 G	2.83 ms	94.6%
4	0	1	173.638 K	0.56 G	2.95 ms	98.5%
4	1	1	379.912 K	1.19 G	1.58 ms	98.7%
**4**	**2**	**1**	**769.320 K**	**2.41 G**	**1.86 ms**	**99.7%**
4	2	2	901.656 K	2.81 G	2.02 ms	99.8%
5	1	1	920.808 K	2.87 G	7.02 ms	99.5%
5	2	1	945.896 K	2.95 G	7.87 ms	99.5%
6	1	1	1.041 M	3.23 G	7.57 ms	99.6%

Significant values are in bold.

In terms of network efficiency, as the network structure deepens, the inference time shows a trend from decline to rise. It shows that a reasonable combination of network modules can not only make the network more powerful but also more efficient. Specifically, compare the LH-ViT(4-0-0) and LH-ViT(4-1-1). LH-ViT(4-1-1) adds 1 Radar-ViT and 1 auxiliary RES-SE on the four-layer pyramid in LH-ViT(4-0-0). Both network parameters and FLOPs are doubled in LH-ViT(4-1-1). LH-ViT(4-1-1) has higher accuracy and less interference time. A similar pattern can also be found in the comparison of LH-ViT(3-0-0) and LH-ViT(3-1-1). It shows that Radar-ViT can help the feature pyramid to make better use of the GPU, making it more efficient to implement a single MDM inference and thus faster.

Finally, the LH-ViT(4-2-1) network, marked in bold in Table 3, is used as a reference structure for subsequent comparison and discussion. The results of the LH-ViT(0-2-1) and LH-ViT(4-0-0) network in Table 3 can be regarded as ablation experiments. It shows the network performance that only includes the feature extractor or the feature enhancement part. The HAR accuracy of the LH-ViT(0-2-1) network without the feature pyramid is only 91.7% and requires 24.29 ms inference time. This shows that Radar-ViT based on the multi-head attention needs MDM feature pre-extraction. Insufficient feature extraction can greatly degrade its performance. Radar-ViT enables important feature attention among pre-extracted rich features. The importance of the attention mechanism has been generally accepted, which also accounts for the performance improvement in the inference efficiency and accuracy of the LH-ViT(4-2-1) network relative to the LH-ViT(4-0-0) network. The results show that the performance of the hybrid network including feature pyramid and Radar-ViT outperforms that of a single network. Compared with LH-ViT(0-2-1)and LH-ViT(4-0-0), the LH-ViT(4-2-1) has improved the accuracy by 8% and 5.1% respectively, and the inference time has been shortened by 22.43 ms and 0.97 ms respectively. It means the LH-ViT(4-2-1) network can achieve more accurate and efficient HAR from MDM.

Table 4 compares the network performance with different $L$ in the Radar-ViT module. Experimental results show that increasing the transformer repetitions does not improve the network performance significantly. Conversely, a bigger L leads to an increase in the parameters and FLOPs, which is not conducive to a lightweight design. At the same time, the inference time also increases. Therefore, a setting of $L=2$ is adopted in the reference LH-ViT(4-2-1) network.

**Table 4:** Comparison of parameters for different numbers of transformers based on the self-established dataset.

$L$	Parameters	FLOPs	Inference time	Accuracy
2	769.320 K	2.41 G	1.86 ms	99.7%
4	943.976 K	2.95 G	4.45 ms	99.8%
6	1.118 M	3.49 G	5.04 ms	99.9%

Table 5 compares the network performance with different convolutional structures. The RES-SE module in the LH-ViT(4-2-1) network is replaced by conventional convolution^11^, transposed convolution^34^, dilated convolution^35^, and group convolution^36^ respectively. The network using the RES-SE module achieves the highest measured parameters and FLOPs, but at the same time, it also has the shortest inference time and highest HAR accuracy. Compared with the best-performing group convolution in the comparison module, the recognition accuracy of the structure using the RES-SE module is improved by 0.9%, and the inference time is shortened by 1.33 ms. It illustrates the superiority of the LH-ViT network based on the RES-SE module for micro-Doppler feature extraction.

**Table 5:** Performance comparison of different convolution structures based on the self-established dataset.

Convolution structure	Parameters	FLOPs	Inference time	Accuracy
Conventional convolution^11^	783.684 K	2.33 G	3.30 ms	95.7%
Transposed convolution^34^	783.437 K	2.33 G	3.27 ms	98.1%
Dilated convolution^35^	783.354 K	2.34 G	3.32 ms	97.8%
Group convolution^36^	743.960 K	2.12 G	3.19 ms	98.8%
RES-SE module	769.320 K	2.41 G	1.86 ms	99.7%

In the comparison of four different attention mechanisms in Table 6, the SE module demonstrates the highest accuracy and the shortest inference time in the micro-Doppler feature extraction.

**Table 6:** Performance comparison of different attention module in RES-SE based on the self-established dataset.

Attention modul	Parameters	FLOPs	Inference time	Accuracy
CA^37^	766.296 K	2.39 G	3.03 ms	99.1%
CBAM^38^	769.472 K	2.41 G	2.03 ms	99.5%
ECA^39^	740.318 K	2.32 G	1.72 ms	99.3%
SE	769.320 K	2.41 G	1.86 ms	99.7%

**Figure 5:**
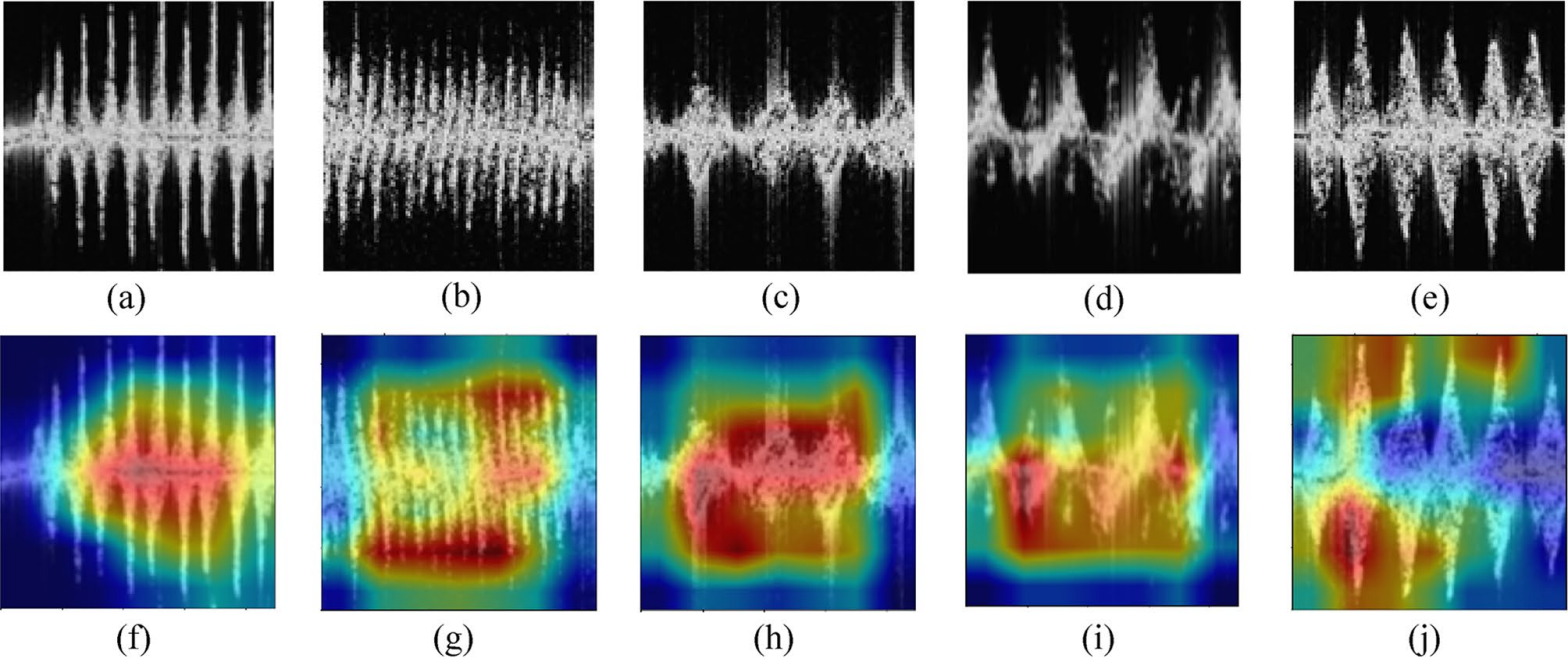
Grayscale images of the five actions along with their heatmaps, (**a**) grayscale image of walking. (**b**) grayscale image of running. (**c**) grayscale image of standing up after squatting down. (**d**) grayscale image of bending. (**e**) grayscale image of turning. (**f**) heatmap of walking. (**g**) heatmap of running. (**h**) heatmap of standing up after squatting down. (**i**) heatmap of bending. (**j**) heatmap of turning.

To better comprehend the role of the attention mechanism in the HAR task based on the radar signals, Fig. 5 depicts the feature region in MDM that the last layer of the attention mechanism focuses on. Heatmaps highlight the regions considered crucial for HAR by the LH-ViT network, facilitating the visual display. The first row displays five grayscale MDM images with activity labels. The second row displays the matching heatmaps for the grayscale MDM image. The red regions on the heatmap indicate the regions that the network prioritizes. The majority of red patches in the attention heatmap are dispersed near endpoints and the Doppler center, reflecting changes in micro-Doppler distributions. It aligns with the Doppler distribution characteristics that can reflect human activities in MDM.

### LH-ViT versus state-of-the-art and literature networks

Table 7 used the state-of-the-art DL networks and the literature networks which have been applied to solve the HAR problem based on radar signals for comparative discussion. The accuracy of these networks was tested on both datasets. The public dataset has a larger input data size and less data volume. Due to the different Doppler scales, the MDMs in the public dataset have lower micro-Doppler features significance. This all increases the difficulty of achieving accurate HAR on the public dataset.

**Table 7:** Comparison of state-of-the-art networks.

Networks	Parameters	FLOPs	Inference time	Accuracy of the public dataset	Accuracy of the self-established dataset
SVM^7^	657.41 K	193.5 M	0.56 ms	59.1%	71.7%
HMM^40^	732.5 K	274.6 G	0.72 ms	60.7%	75.4%
ShuffleNet^41^	346.917 K	426.3 M	1.38 ms	88.6%	95.5%
EfficientNet^42^	4.01 M	398 M	1.48 ms	88.4%	98.8%
LSTM^15^	11.6 M	7.88 G	38.48 ms	60.3%	75.3%
GRU^43^	8.76 M	5.91 G	36.43 ms	63.9%	96.9%
DeiT^24^	5.679 M	1.08 G	1.40 ms	83.9%	98.7%
CrossViT^44^	6.649 M	1.29 G	1.90 ms	87.5%	87.8%
MobileViT^26^	1.27 M	1.44G	15.17 ms	91.3%	98.9%
LSTM-BiLSTM^17^	282.285 K	10.6 G	32.30 ms	76.1%	96.3%
Stack3-LSTM^18^	3.08 M	446.47 M	5.17 ms	72.3%	95.4%
Mobile-RadarNet^20^	241.1 K	**3.11 M**	2.61 ms	85.7%	95.6%
CLA^25^	**97.57 K**	12.57 M	**0.38 ms**	89.1%	97.1%
Slice-VIT^45^	85 M	16.86 G	38.47 ms	86.4%	99.1%
LH-ViT(4-2-1)	769.32 K	2.41 G	1.58 ms	**92.1%**	**99.7%**

Significant values are in bold.

The HAR accuracy of SVM and HMM is relatively low. ShuffleNet^41^ and EfficientNet^42^ are convolutional neural networks. Among them, Shufflenet has fewer parameters, but lower accuracy. The parameter amount of Efficientnet has reached about 4M, and its accuracy rate is high. The inference time of both networks above is within 1.5 ms. LSTM^15^ and GRU^43^ are sequential networks of RNN variants, in which GRU has a higher accuracy rate. The main problem with this type of network is the parameter quantity and interference efficiency introduced by the network complexity. DeiT^24^, CrossViT^44^ and MobileViT^26^ are three lightweight ViT network examples with good performance. DeiT has a smaller number of parameters, Flops, and shorter inference time. The accuracy of DeiT is higher on the self-established dataset, but lower on the public dataset. MobileViT has better performance but longer inference time.

Stack3-LSTM^18^ and LSTM-BiLSTM^17^ realize HAR in the form of a hybrid network considering the timing correlation characteristics of radar human motion signals. Both networks achieved over 95% HAR accuracy on the self-established dataset. However, similar to LSTM and GRU, such networks’ accuracy comes at the expense of a huge number of network parameters and time overhead, and both networks’ performance shows a sharp decrease in the public dataset. Mobile-RadarNet^20^ has the smallest FLOPs, but its accuracy is not competitive among the networks in Table 7. CLA^25^ has the fewest parameters and the fastest inference time, and its accuracy is also at a good level in both datasets. Although Slice-VIT^45^ makes ViT better adaptable in solving radar-based HAR through slice preprocessing, the complexity and efficiency of this network are still key issues to be solved.

The LH-ViT proposed serves as a lightweight hybrid network of convolution and ViT. The highest accuracy is achieved on both datasets. Moreover, the amount of parameters is the smallest among the ViT-type networks, and the inference time is also at a relatively fast level. The above results illustrate the excellent performance of the LH-ViT network as well as its good adaptability and robustness.

**Figure 6:**
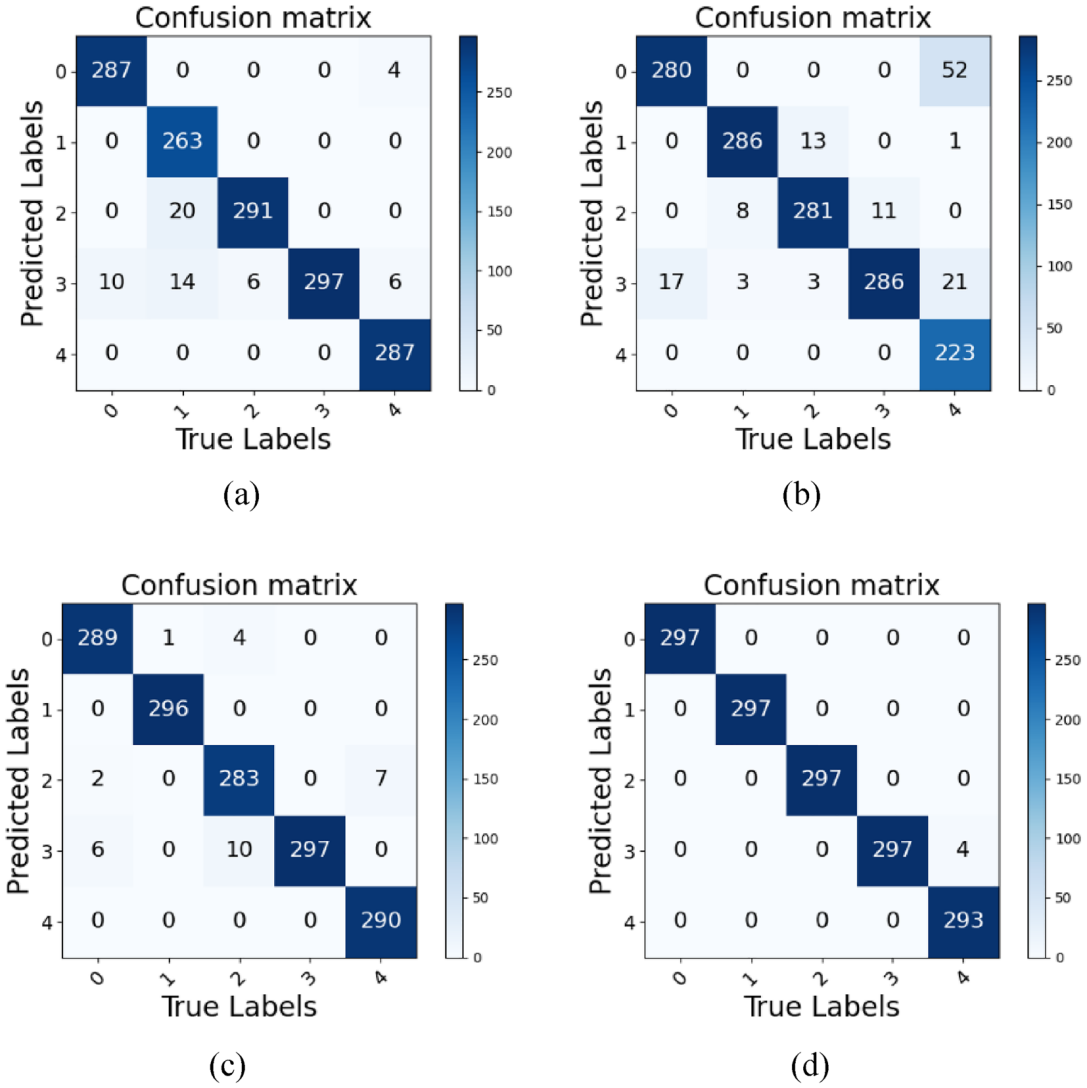
Comparison of confusion matrices in different networks, (**a**) ShuffleNet. (**b**) GRU. (**c**) DeiT. (**d**) LH-ViT(4-2-1).

The confusion matrix illustrates the specific recognition results of four lightweight network models using 297 images for each activity, as shown in Fig. 6. LH-ViT only had four images misrecognized in the turning category for bending. Unlike vision-based HAR, radar-based HAR is achieved through the time-dependent variation in the micro-Doppler components introduced by limb movements, so the frequency characteristics of human movements determine the degree of different activity similarity. Human activities which exhibit similar features in the Doppler domain along slow time will lead to recognition errors. Despite this, the performance of LH-ViT is the best among the four networks.

Subject-independent split can reflect the individual differences sensitivity and the generalization performance of the proposed network. The public dataset contains 20 people’s radar data of activities, of which 16 individuals are used for training and 4 for testing. For the self-established dataset, 8 people’s data are used for training and 2 for testing. Tables 8 and 9 show the results of the subject-independent split experiment under different datasets respectively. The accuracy of LH-ViT(4-2-1) is only reduced by 0.4% and 0.2% respectively in the public dataset and the self- established dataset. These results are better than the MobileViT in both individual activity accuracy and comprehensive accuracy. It shows that the LH-ViT network proposed in this paper can well adapt to the individual differences and achieve high performance radar-based HAR through accurate Micro-Doppler feature extraction.

**Table 8:** Subject-independent split experiment based on the public dataset.

Networks	Accuracy						
	Label (0)	Label (1)	Label (2)	Label (3)	Label (4)	Label (5)	ALL
MobileViT	91.5%	91.3%	91.4%	91.5%	91.5%	90.2%	90.9%
LH-ViT(4-2-1)	**92.4%**	**92.5%**	**92.5%**	**92.4%**	**92.2%**	**91.1%**	**91.7%**

Significant values are in bold.

**Table 9:** Subject-independent split experiment based on the self-established dataset.

Networks	Accuracy					
	Label (0)	Label (1)	Label (2)	Label (3)	Label (4)	ALL
MobileViT	98.4%	98.5%	98.4%	98.5%	98.7%	98.6%
LH-ViT(4-2-1)	**99.4%**	**99.6%**	**99.4%**	**99.5%**	**99.6%**	**99.5%**

Significant values are in bold.

## Conclusion

This paper developed a lightweight hybrid Vision Transformer network for HAR based on radar's micro-Doppler features. After preprocessing, the network can obtain the recognition accuracy of 99.7% in the self-established dataset and 92.1% in the public dataset respectively. We investigated the performance of the proposed network under various architectures and obtained the optimal structure. The optimal structure was compared with other widely used networks as well as HAR networks in the literature and showed performance advantages. The proposed network satisfies the accuracy and real-time requirements for HAR and is promising for embedded applications. This work is only used for single-action recognition, and the collection scenario is relatively ideal. In the future, we plan to improve and expand the number and type of data sets, develop the radar signal processing algorithms, and optimize the deep learning network structure to improve radar-based HAR performance in the face of complex and continuous human activities.

## Data Availability

The datasets used and/or analyzed during the current study available from the first author on reasonable request.
